# Supplementary material for: Surface Ligand Influences the Cu Nanoclusters as a Dual Sensing Optical Probe for Localized pH Environment and Fluoride Ion
Source: Nanomaterials (Basel). 2023 Jan 28;13(3):529. doi: 10.3390/nano13030529 (PMC9919789; doi:10.3390/nano13030529)
Supplement: Supplementary file 1 [file nanomaterials-13-00529-s001.zip › nanomaterials-2133769-supplementary.pdf]

nanomaterials

*Supplementary Material*

# Surface Ligand Influences the Cu Nanoclusters as a Dual Sensing Optical Probe for Localized pH Environment and Fluoride Ion

**Kumar Babu Busi [1], Subhalaxmi Das [1], Mathangi Palanivel [2], Krishna Kanta Ghosh [2], Balázs Gulyás [2,3,4], Parasuraman Padmanabhan [2,3,*] and Sabyasachi Chakrabortty [1,*]**

[1] Department of Chemistry, SRM University, AP—Andhra Pradesh, Guntur, Andhra Pradesh 522 240, India
[2] Lee Kong Chian School of Medicine, Nanyang Technological University Singapore, 59 Nanyang Drive, Singapore 636921, Singapore
[3] Cognitive Neuroimaging Centre, Nanyang Technological University, 59 Nanyang Drive, Singapore 636921, Singapore
[4] Department of Clinical Neuroscience, Karolinska Institute, 17176 Stockholm, Sweden
* Correspondence: ppadmanabhan@ntu.edu.sg (P.P.); sabyasachi.c@srmap.edu.in (S.C.)

## Additional results

1. Emission spectra of BSA-Cu NCs at different excitation wavelength
2. Radiative decay results of BSA-Cu NCs
3. Zeta potential values of BSA-Cu NCs
4. pH reversibility experiment
5. Optical characteristics of Cys-Cu NCs
6. Absorbance spectra of Cys-Cu NCs with different buffer solutions
7. PL intensity spectra of Cys-Cu NCs with different buffer solutions
8. Zeta potential measurement of Cys-Cu NCs with different buffer solutions
9. Average size analysis of Cys-Cu NCs
10. Limit of detection measurements for $F^-$ ion detection
11. $F^-$ ion detection in real samples

**1. Emission spectra of BSA-Cu NCs at different excitation wavelength**

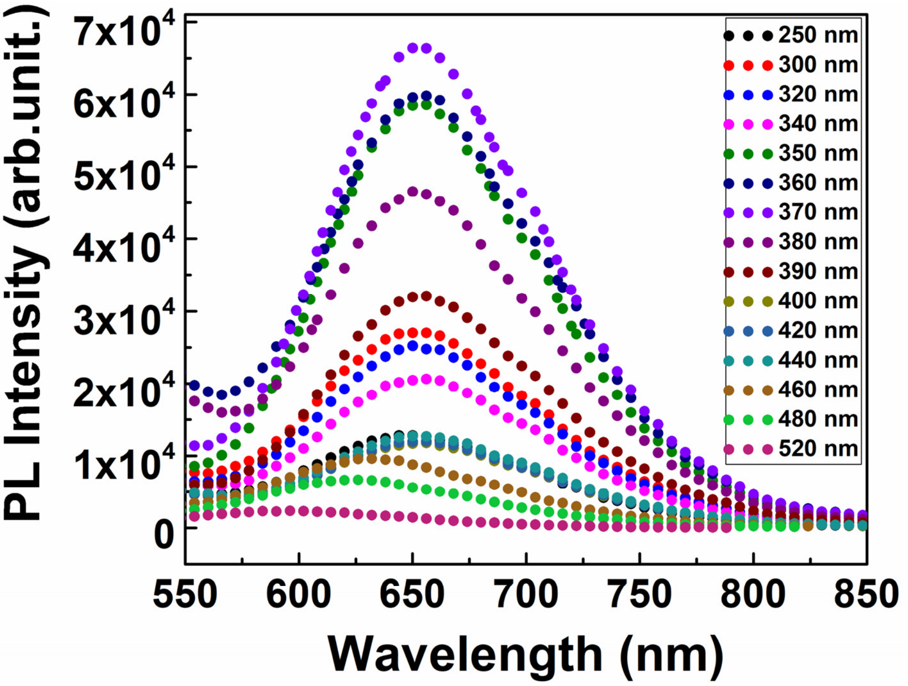


**Figure S1.** The photoluminescence (PL) intensity recorded for the as-prepared BSA-Cu NCs at various excitation wavelengths, with the highest red colour emission maximum observed at 652 nm and the excitation maximin reported at 370 nm.

**2. Radiative decay results of BSA-Cu NCs**

**Table S1.** The time resolved PL measurement of BSA-Cu NCs with the combination of various buffers and control sample as well. The percentage of the relative amplitudes (A1, A2, A3) are having direct correlation with radiative decay patterns, where the τ1, τ2 and τ3 represent the nonradiative decay related to the present of defects, radiative decay, and Auger decay process respectively.

| Sample name | Relative Amplitude | | | PL decay | | | Average Lifetime |
|---|---|---|---|---|---|---|---|
| | $A_1$ | $A_2$ | $A_3$ | $\tau_1$ | $\tau_2$ | $\tau_3$ | |
| **Control** | 5.48 | 90.35 | 4.17 | 1.47E-07 | 1.91E-06 | 2.41E-09 | **1.74E-06** |
| **pH—5** | 9.63 | 85.48 | 4.89 | 4.14E-07 | 3.36E-06 | 1.76E-09 | **2.90E-06** |
| **pH—7** | 10.12 | 84.18 | 5.7 | 3.87E-07 | 2.59E-06 | 1.79E-09 | **2.22E-06** |
| **pH—11** | 11.56 | 79.76 | 8.68 | 4.12E-07 | 3.51E-06 | 8.68E-09 | **2.85E-06** |

### 3. Zeta potential analysis of BSA-Cu NCs

**Table S2.** The zeta potential measurement of BSA-Cu NCs to determine the surface charge of BSA-Cu NCs with the combination of different buffers and control sample as well.

| Sample name | Measurement 1 | Measurement 2 | Measurement 3 | Average surface charge (eV) |
|---|---|---|---|---|
| **Control** | -19.4 | -18.6 | -20.2 | **-19.4** |
| **pH—5** | -9.98 | -12.6 | -11.6 | **-11.3** |
| **pH—7** | -8.77 | -9.38 | -7.56 | **-8.57** |
| **pH—11** | -9.67 | -8.06 | -9.21 | **-8.98** |

### 4. pH reversibility experiment

In brief, 50 μL of the as synthesized BSA-Cu NCs were mixed with 50 μL of pH–7 and pH–11 buffer solutions and the vigorously shaked at room temperature for 10 minutes. The fluorescence spectra of these mixtures were collected by TECAN microplate reader. The pH reversibility experiments were carried out by altering the pH with the addition of previously prepared universal buffer solutions. However, the pH reversibility experiment protocol was clearly stated in the figure below, and the same procedure was followed for the green emitting Cu NCs as well.

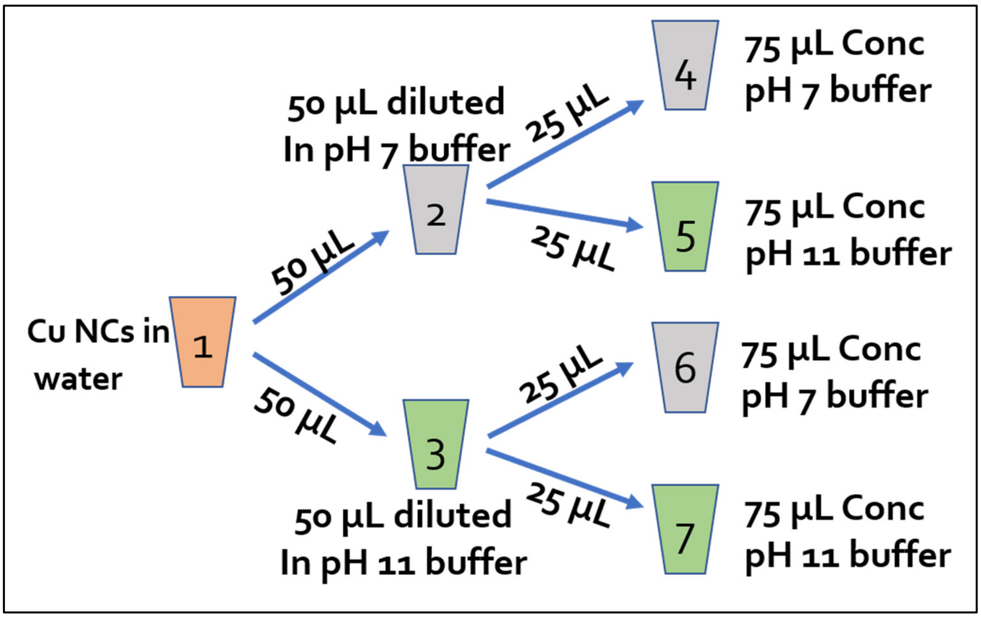


**Figure S2.** The pH reversibility studies were carried out using the above-mentioned schematic methodology for the two different colours emitting Cu NCs.

## 5.Optical properties of Cys-Cu NCs

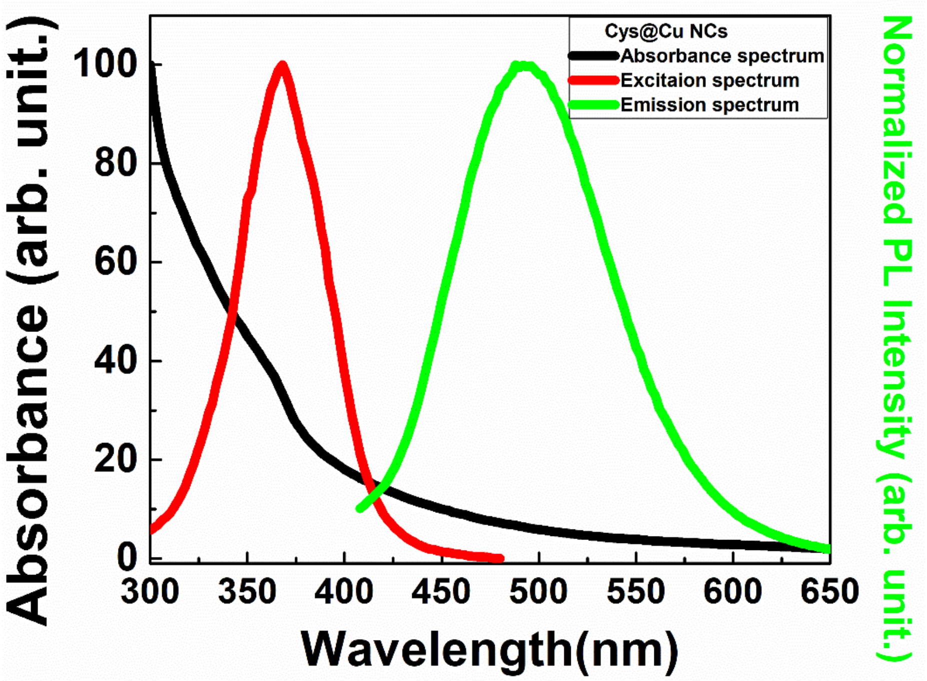


**Figure S3.** The optical characteristics of Cu NCs that produced green colour emission and were stabilized with a single amino acid cysteine. Due to the lack of a surface plasmon resonance peak, the absorbance spectrum (black curve) suggests that bigger size particles are absent. The strongest excitation maximum (red curve) was recorded at 370 nm, whereas the highest green colour emission intensity (green curve) was seen at 492 nm. .

## 6. Absorbance spectra of Cys-Cu NCs with different buffer solutions

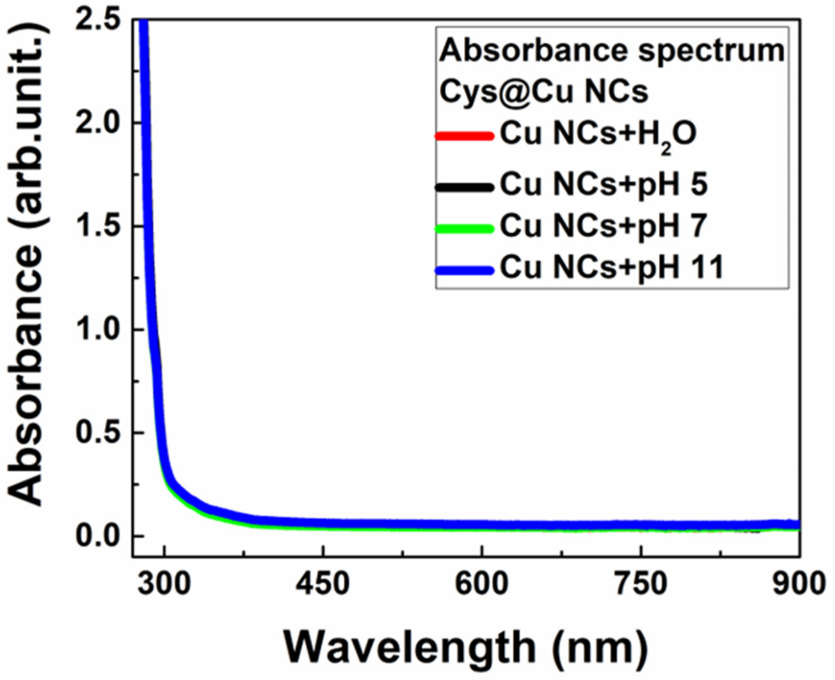


**Figure S4.** The absorbance spectra of Cys-Cu NCs were unable to exhibit much difference due to their tiny size with the addition of different buffer solutions.

## 7. PL intensity spectra of Cys-Cu NCs with different buffer solutions

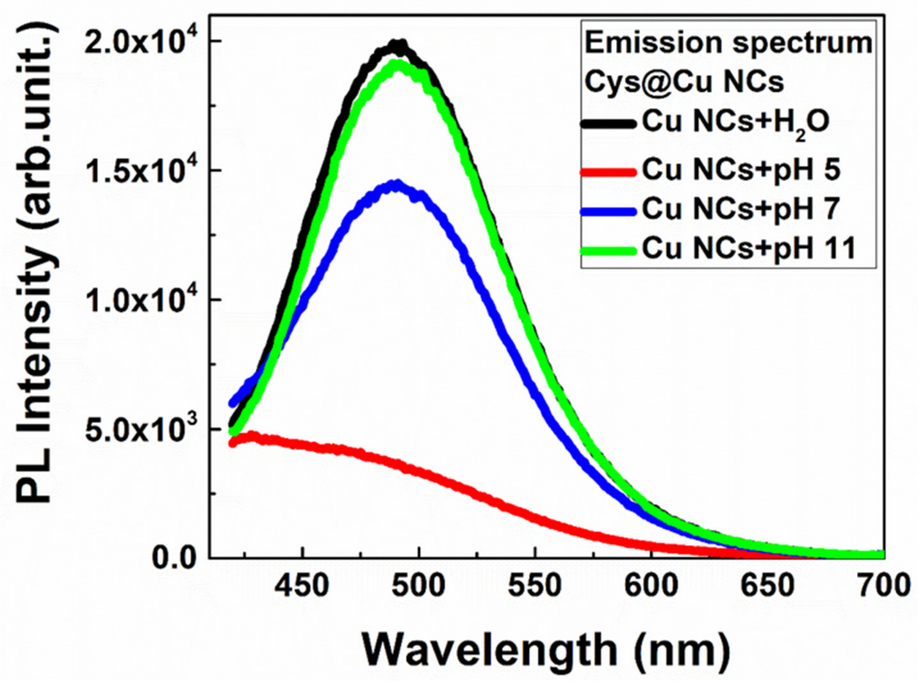


**Figure S5.** Green colour emitting Cys-Cu NCs showed a remarkable variation in the rate of electron and hole recombination in their PL intensity spectra. The addition of pH-11 buffer maintains the PL intensity same as control, however the addition of pH-5 and pH-7 buffers causes the fluorescence to dynamically quench.

## 8. Zeta potential measurement of Cys-Cu NCs with different buffer solutions

**Table S3.** The zeta potential measurement was performed to evaluate the surface charge of Cys-Cu NCs with the addition of three different phosphate buffer solutions pH 5, 7, 11and the control sample.

| Sample name | Measurement 1 | Measurement 2 | Measurement 3 | Average surface charge (eV) |
|---|---|---|---|---|
| **Control** | -35.7 | -37.4 | -39.3 | **-37.4** |
| **pH—5** | 6.26 | 6.18 | 6.62 | **6.35** |
| **pH—7** | -7.36 | -7.3 | -11.2 | **-8.62** |
| **pH—11** | -32.7 | -34.9 | -37 | **-34.8** |

## 9. Average size analysis of Cys-Cu NCs

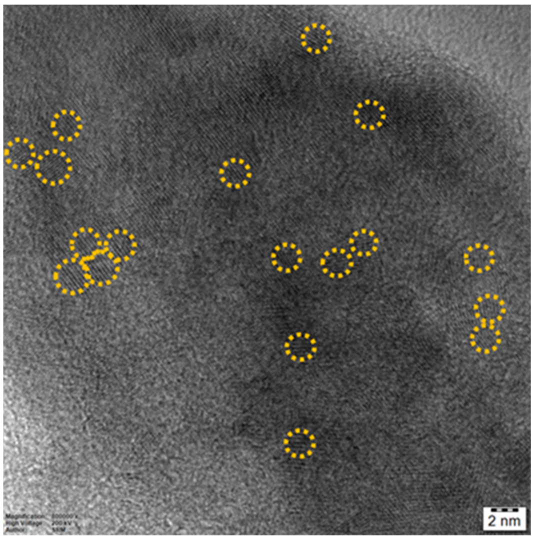

**Figure S6.** The HRTEM image of cysteine stabilised Cu NCs revealed the Cu (111) plane with 2.01 A° of d-spacing and an average size of ~1.98±0.2 nm.

### 10. Limit of detection measurements for $F^-$ ion detection

**Table S4.** The detection limit calculations for red and green colour emitting Cu NCs for $F^-$ ion detection.

| | Red Cu NCs | Green Cu NCs |
|---|---|---|
| **Linear Regression** | y = 0.4351x + 4.7656 | y = 0.4557x + 1.0137 |
| **$R^2$ value** | 0.981589608 | 0.991071498 |
| **Standard Error** | 0.977661853 | 0.709657998 |
| **Slope** | 0.435064938 | 0.455663508 |
| **Formula = 3σ/K** | 3*0.977661853/0.4351 | 3*0.709657998/0.4557 |
| **LOD** | **6.741489144** | **4.672250374** |

### 11. $F^-$ ion detection in real samples

**Table S5.** Analytical results for the detection of $F^-$ ion in real time samples including tap water, lake water and river water.

| | Sample name | Spiked (μM) | Detected (μM) | Recovery (%) |
|---|---|---|---|---|
| BSA-Cu NCs | Tap water | 10 | 9.695468 | 96.95468 |
| | Lake water | 10 | 9.805324 | 98.05324 |
| | River water | 10 | 9.92029 | 99.2029 |
| Cys-Cu NCs | Tap water | 10 | 9.941092 | 99.41092 |
| | Lake water | 10 | 10.37844 | 103.7844 |
| | River water | 10 | 10.06426 | 100.6426 |
